# Supernatants from stored platelet concentrates modulate the inflammatory response in platelet-depleted blood ex vivo

**DOI:** 10.3389/fimmu.2026.1928875

**Published:** 2026-10-05

**Authors:** Linnea I. Andersson, Dick J. Sjöström, Nahreen Tynngård, Camilla Mohlin, Huy Quang Quach, Ivar Tjernberg, Sofia Ramström, Tom Eirik Mollnes, Per H. Nilsson

**Affiliations:** 1 Linnaeus Centre for Biomaterials Chemistry, Linnaeus University, Kalmar, Sweden; 2 Department of Chemistry and Biomedicine, Linnaeus University, Kalmar, Sweden; 3 Department of Biomedical and Clinical Sciences, Linköping University, Linköping, Sweden; 4 Healthcare Research Strategy Department, Region Östergötland, Linköping, Sweden; 5 Mayo Clinic Vaccine Research Group, Mayo Clinic, Rochester, MN, United States; 6 Department of Clinical Chemistry and Transfusion Medicine, Region Kalmar County, Kalmar, Sweden; 7 Department of Biomedical and Clinical Sciences, Division of Inflammation and Infection, Linköping University, Linköping, Sweden; 8 Cardiovascular Research Centre, School of Medical Sciences, Faculty of Medicine and Health, Örebro University, Örebro, Sweden; 9 Department of Immunology, Oslo University Hospital and University of Oslo, Oslo, Norway; 10 Research Laboratory, Nordland Hospital, Bodo, Norway

**Keywords:** cytokines, inflammation, platelets, thromboinflammation, whole blood

## Abstract

**Introduction:**

Platelets contain a wide range of bioactive mediators, many of which accumulate during storage of platelet concentrates and have immunomodulatory properties. Our objective was to investigate how mediators derived from stored platelet concentrates modulate the acute inflammatory response using an ex vivo model.

**Methods:**

Platelet-depleted whole blood from healthy donors (n=8) was supplemented with pooled platelet concentrate supernatants generated from six platelet concentrates sampled on storage days 1, 3, 5, and 7, and incubated with or without heat-inactivated *Escherichia coli* for four hours at 37 °C. Inflammatory responses were assessed by measuring complement activation, platelet-derived mediators, and cytokines.

**Results:**

In the absence of *E. coli*, addition of platelet concentrate supernatants increased the levels of 24 of 32 measured inflammatory mediators. In contrast, under *E. coli*-stimulated conditions, the supernatants increased 10 markers and attenuated 14 markers, including the key pro-inflammatory cytokines IL-1β, IL-6, and TNF. The attenuation of these cytokines ranged from 25-43%, whereas several other mediators were unchanged or increased. The majority of biomarkers showed limited differences across the investigated storage time points.

**Conclusions:**

Our findings demonstrate that soluble factors released from platelet concentrates can modulate inflammatory responses in platelet-depleted blood in an ex vivo setting, with context-dependent, bidirectional, and selective immunomodulatory effects.

## 1 Introduction

Platelets are essential for hemostasis and play a key role in immune regulation by releasing a wide range of bioactive mediators upon activation. These mediators, stored in platelet granules, contribute not only to thrombosis and wound healing but also to inflammation and antimicrobial host defense (1–3). Platelet transfusions are primarily administered to prevent or control bleeding in patients with thrombocytopenia, most commonly in hematology and oncology, but also in cardiac surgery and intensive care settings (4–7). In these clinically vulnerable populations, and particularly in patients with infection, trauma, or treatment-related tissue injury, platelet transfusion is not only a hemostatic intervention but also a source of soluble factors that may shape the recipient’s immune response. Pre-existing inflammatory activation may amplify transfusion-related effects and contribute to adverse outcomes.

Platelet transfusion has been associated with both beneficial (8, 9) and adverse immunological effects (10). In particular, transfusion may act as a “second hit” in patients with pre-existing inflammatory activation, contributing to complications such as febrile and allergic reactions, transfusion-related acute lung injury (TRALI) and transfusion-associated circulatory overload (TACO) (11, 12). These complications reflect the interaction between the recipient’s inflammatory state and the biological properties of the transfused product (13, 14), including plasma content and manufacturing approach, which have been linked to different adverse reaction rates (15, 16). Platelet concentrates undergo storage-associated biochemical and immunological changes, however, the evidence on the role of storage duration and product characteristics remains heterogeneous (17–19).

Platelet concentrates for transfusion are generally stored for up to 5–7 days, depending on country-specific regulations (19). During storage, platelets can develop “storage lesions”, including activation and release of damage-associated molecular patterns (DAMPs), attenuated responsiveness to agonists, metabolic changes, shedding of platelet-derived microvesicles, and accumulation of complement activation fragments into the storage solution (20–22). These storage-related changes may impact the inflammatory potential of the product after transfusion. Our previous work has shown that platelet storage is accompanied by complement activation and platelet functional changes (22, 23), indicating that storage conditions may influence downstream immune responses. Innate immune responses are initiated when molecular patterns associated with pathogens or tissue damage activate pattern recognition systems, including the complement cascade and Toll-like receptors (TLRs). These pathways are central drivers of cytokine release and inflammatory amplification, and their dysregulation can lead to systemic inflammation and organ dysfunction (24–27).

The present study examined whether soluble mediators derived from stored platelet concentrates modulate innate immune activation in platelet-depleted whole blood ex vivo. By focusing on platelet supernatants, this model isolates the contribution of soluble storage-derived factors, while not capturing receptor-mediated interactions or the full cellular complexity of intact transfusion products. Heat-inactivated *E. coli* was used as a standardized innate immune stimulus, enabling us to examine whether platelet-derived soluble factors modify responses under conditions of acute immune activation. Although this stimulus does not model the full spectrum of clinically relevant inflammatory triggers, it addresses whether storage-derived soluble factors from platelet concentrates can shape inflammatory responses in a controlled ex vivo system and if storage duration alters that effect under basal and stimulated conditions. To comprehensively evaluate the immunomodulatory effects of platelet concentrate supernatants, a biomarker panel was selected to capture pathways involved in innate immune activation. The panel included markers of platelet activation, complement activation, and leukocyte-derived inflammatory mediators (22, 28–30), thereby enabling assessment of both mediators present in platelet concentrate supernatants and secondary inflammatory responses induced in recipient blood.

## 2 Materials and methods

### 2.1 Platelet concentrate preparation

Whole blood donations were collected from healthy volunteers at Kalmar Region Hospital, Sweden. Each whole blood unit was processed into an interim platelet unit, plasma, and erythrocytes using the Reveos^®^ system (Terumo BCT, Larne, UK). Interim platelet units from 3 to 6 donors were pooled using a leukocyte filter set (Terumo BCT) and suspended in PAS-E (SSP+, Macopharma, Mouvaux, France) to produce a platelet concentrate. The platelet concentrates were evaluated for hematological parameters using an automated hematology instrument (Sysmex, Kobe, Japan). Leukocytes were counted by flow cytometry (BD FACSCanto II, Franklin Lakes, NJ). Bacterial contamination was screened using the BACT/ALERT^®^ VIROTU^®^ blood culture detection system (BioMérieux SA RCS, Lyon, France). Finally, the platelet concentrates underwent gamma irradiation at 25 Gy (TrueBeam, Varian, Palo Alto, CA). Standard manufacturer’s instructions and protocols were followed for each commercial platform used.

### 2.2 Storage, sampling and preparation of platelet supernatants

Six platelet concentrates were stored with constant agitation (Meloc Engineering Corp., Glendale, CA) in a 22°C incubator (Forma platelet incubator, Thermo Electron Corporation, Waltham, MA) for their seven-day shelf life. Samples of 30 mL were aseptically drawn on days 1, 3, 5 and 7 into a sampling bag (Macopharma). Each sample was transferred to a 50 mL polypropylene tube (Sarstedt, Nümbrecht, Germany), recalcified to a final Ca^2+^ concentration of 25 mM to neutralize the citrate in PAS-E solution, and supplemented with the thrombin inhibitor lepirudin (Refludan^®^, Celgene, Uxbridge, UK) at a concentration of 50 μg/mL to prevent clotting. Each sample contained between 746 and 931×10^6^ platelets per mL (mean ± SD: 846 ± 60 × 10^6^/mL). The samples were centrifuged at 3000 × g for 20 minutes at 22 °C, after which the supernatants were collected, snap-frozen in liquid nitrogen, and stored at -80°C. Once supernatants from all six platelet concentrates were processed, they were thawed on slushed ice and pooled into platelet concentrate supernatant pools (PCSPs) according to storage day (Days 1, 3, 5, or 7) (**Figure 1**). The pooled samples were mixed, snap-frozen in liquid nitrogen, and stored at -80°C. This pooling strategy enabled assessment of the biological effects of platelet concentrate supernatants across multiple recipient blood donors. However, it precluded assessment of variability between individual platelet concentrate units.

**Figure 1:**
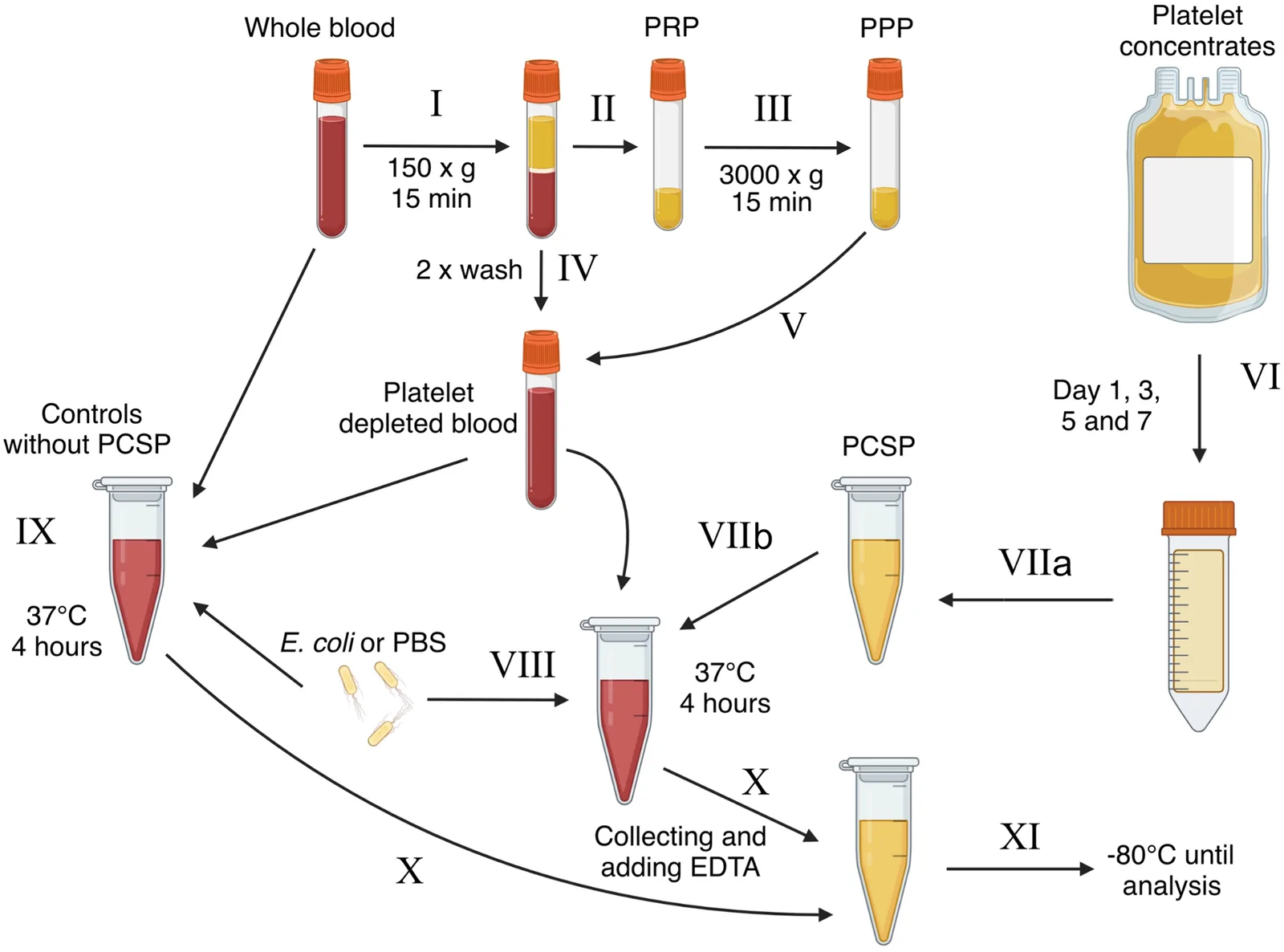
Platelet depletion protocol and experimental setup. Lepirudin (50 μg/mL) anticoagulated whole blood (n=8) was centrifuged (I) to obtain platelet-rich plasma (PRP). PRP was separated from the cells (II) and centrifuged further (III) to obtain platelet-poor plasma (PPP). The cells were washed two times (IV) and the PPP was reintroduced (V) to the cells to create platelet-depleted blood. Platelet concentrates (n=6) were stored at 22 °C for seven days. At Day 1, 3, 5, and 7, a sample was taken aseptically (VI) and centrifuged to remove platelets. The supernatants from all six platelet concentrates were pooled into one platelet concentrate supernatant pool (PCSP) (VIIa). The PCSPs were added to platelet-depleted blood (VIIb) and subsequently incubated with or without *E. coli* (10^7^/mL) for 4 hours at 37 °C (VIII). Whole blood was incubated in parallel with or without *E. coli* (IX). EDTA was added after the incubation and the blood was centrifuged to plasma (X) and stored at -80 °C until analysis (XI). Created in BioRender. Nilsson, PH (2026). https://BioRender.com/kyekh1o.

### 2.3 Platelet depletion of whole blood

A slightly modified protocol from Quach et al. (30) was used to deplete platelets from whole blood (**Figure 1**). The procedure was optimized in our laboratory using whole blood from six individual donors. We achieved a mean platelet depletion of 96.6% (SD: 2.4%), while retaining an average of 69.2% (SD: 14.2%) of the leukocytes, as confirmed using a hematology analyzer (SweLab Alfa, Boule Diagnostics, Spånga, Sweden). Blood was collected from healthy volunteers (n=8) and supplemented with the highly specific thrombin inhibitor lepirudin (Refludan^®^, final concentration of 50 μg/mL) as an anticoagulant. To deplete platelets, whole blood was centrifuged at 150 × g for 15 minutes at 22 °C with no brake. The top layer, containing platelet-rich plasma (PRP), was isolated and immediately centrifuged at 3000 × g for 15 minutes at 22 °C to obtain platelet-poor plasma (PPP). The bottom layer, containing erythrocytes and leukocytes, was washed twice by adding sterile PBS equal to the volume of the removed plasma, centrifuging at 150 × g for 15 minutes at 22 °C, and discarding the top layer containing PBS along with residual platelets and plasma. After the final wash cycle, the PPP was reintroduced to the cells, forming platelet-depleted blood.

### 2.4 Incubation of platelet supernatants with platelet-depleted blood

Platelet-depleted blood (375 μL) was mixed with 125 μL of the PCSP from days 1, 3, 5 and 7 (**Figure 1**). The same eight platelet-depleted blood donors were used throughout the study and were exposed to all four storage-day platelet concentrate supernatant pools. The samples were then incubated, either with or without heat-inactivated *E. coli* (strain LE392, ATCC 33572, American Type Culture Collection, Manassas, VA), at a concentration of 1.0 × 10^7^ bacteria per milliliter. This strain of *E. coli* at this concentration was used to stimulate an innate immune response due to its dual ability to initiate toll-like receptor- and complement activation (29). After four-hour incubation at 37 °C with continuous rolling, EDTA was added to a final concentration of 10 mM, and the tubes were centrifuged at 3000 × g for 15 minutes at 4°C. The plasma supernatants were collected and stored at -80°C for later biomarker analysis.

### 2.5 Biomarker analysis

Complement activation markers, including C3-activation (C3bc) and the soluble terminal C5b-9 complement complex (TCC), were quantified by ELISA as previously described (31). Platelet activation markers, including NAP-2, PF4, thrombospondin-1 (TSP-1), and transforming growth factor beta 1 (TGF-β1), were analyzed using DuoSet^®^ ELISAs (R&D Systems, Minneapolis, MN). Absorbance was measured using a Tecan Sunrise plate reader (Tecan Nordic, Stockholm, Sweden) and Magellan software (version 7.1, Tecan Nordic) was used for data analysis. Cytokines, including interleukins (IL), chemokines, interferons and growth factors (IL-1β, IL-1ra, IL-2, IL-4, IL-5, IL-6, IL-7, IL-8, IL-9, IL-10, IL-12, IL-13, IL-15, IL-17, bFGF-2, eotaxin, G-CSF, GM-CSF, IFN-γ, IP-10, MCP-1, MIP-1α, MIP-1β, PDGF, RANTES, TNF, and VEGF), were quantified using a human 27-plex kit (Bio-Rad, Hercules, CA) analyzed on a Bio-Plex MAGPIX multiplex system (Bio-Rad). The data was analyzed using Bio-Plex Manager 6.1 (Bio-Rad). All markers except IL-7 were detected at measurable levels. The selected biomarker panel was chosen to cover key pathways relevant to platelet storage lesions and innate immune activation, including platelet activation, complement activation, and leukocyte-derived inflammatory responses. Values at or above the assay limit of detection (LOD) were included in the analysis (**Supplementary Table S1**). Values below the LOD were assigned a value equal to one-half of the assay-specific LOD to enable inclusion in subsequent statistical analyses.

### 2.6 Data presentation and Statistical analysis

Data analysis, statistical comparison, and data visualization were performed using GraphPad Prism (San Diego, CA) version 10 for Mac. Activation marker levels between whole blood and platelet-depleted blood were compared using a paired Student’s *t-test*. The effects of adding PCSPs to platelet-depleted blood were assessed by repeated measures one-way ANOVA with Dunnett’s correction for multiple comparisons, using platelet-depleted blood as the control. A p-value <0.05 was considered statistically significant. The sample size was based on previous experience with this ex vivo whole-blood model and historical datasets generated using comparable inflammatory stimuli and biomarker analyses (32, 33). Normality was assessed using the Shapiro-Wilk test. While some biomarkers showed deviations from normality in individual experimental conditions, repeated-measures ANOVA was retained because the majority of biomarkers (28 of 32) demonstrated distributions consistent with normality across at least eight of twelve experimental conditions. Also, repeated-measures ANOVA is generally considered robust to moderate departures from normality in balanced repeated-measures designs (34).

## 3 Results

### 3.1 PCSP addition to platelet-depleted blood increased complement activation product C3bc

Complement activation, measured by C3bc (**Figure 2A**) and TCC (**Figure 2B**), was significantly reduced in platelet-depleted blood compared with whole blood under both experimental conditions (p<0.05-p<0.01). In the absence of *E. coli*, C3bc and TCC levels were reduced by 35% (p<0.01) and 58% (p<0.01), respectively. Under *E. coli* stimulation, C3bc and TCC levels were reduced by 52% (p<0.01) and 35% (p<0.05), respectively. Supplementation with Day 7 PCSPs significantly increased C3bc levels by 43% (p<0.05) in the absence of *E. coli* and by 47% (p<0.05) in the presence of *E. coli*.

**Figure 2:**
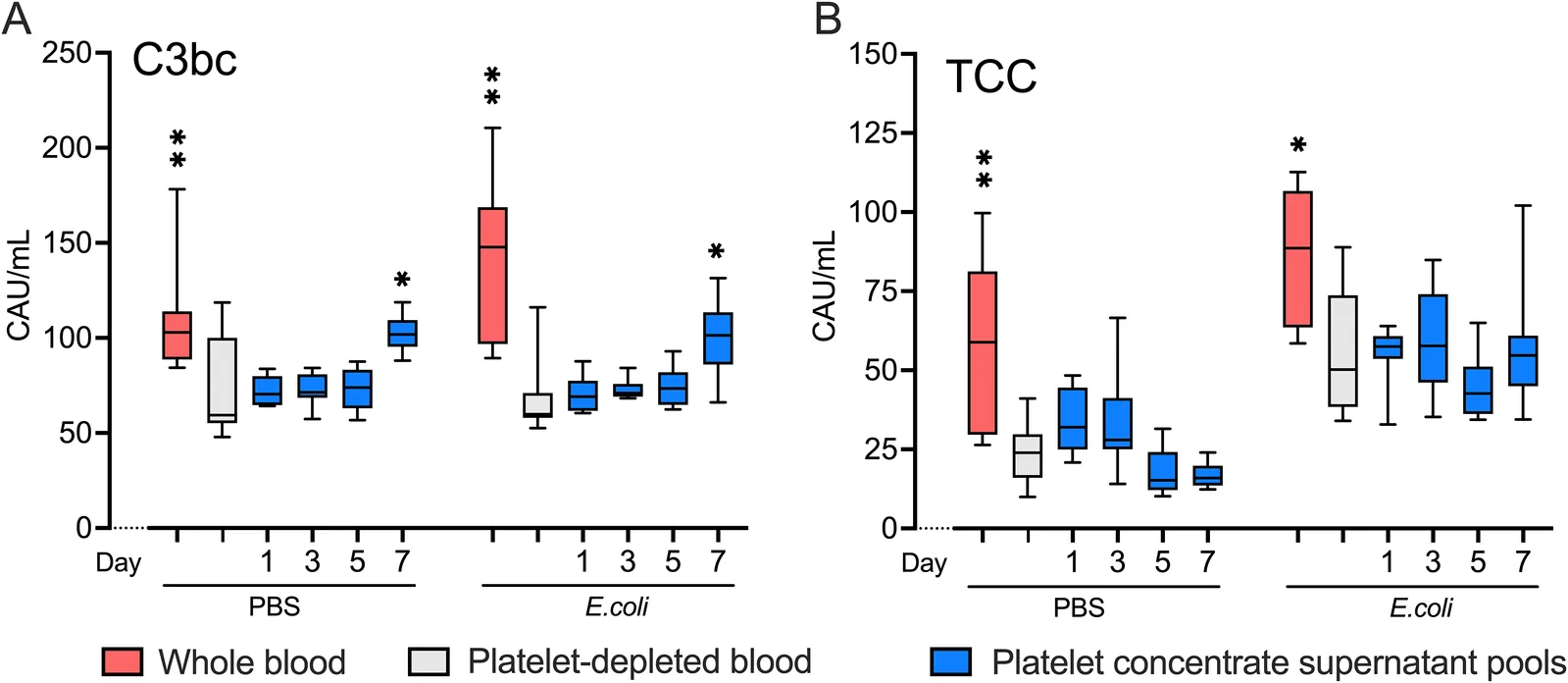
Complement activation in platelet-depleted blood supplemented with supernatants from platelet concentrates (PCSP). Whole blood (red), platelet-depleted blood (gray) and platelet-depleted blood supplemented with supernatants from platelet concentrates (blue) stored for 1, 3, 5 and 7 days was incubated for four hours at 37 °C with PBS or *E. coli* (10^7^/mL), as indicated. Complement activation was analyzed as C3bc **(A)** and TCC **(B)** with ELISA, expressed as complement activating units (CAU) per mL. Data are shown in box (25th to 75th percentiles) and whiskers (min to max) plots, (n=8). Differences between whole blood and platelet-depleted blood were analyzed with a paired Student´s *t-test.* Any effects of PCSP addition were analyzed with a repeated measures one-way ANOVA with Dunnett’s correction for multiple tests using platelet-depleted blood as the control. Statistical significance is shown as *p<0.05, **p<0.01.

### 3.2 PCSP addition to platelet-depleted blood substantially increased platelet activation markers

All assessed platelet activation markers were significantly lower in platelet-depleted blood compared with whole blood, confirming successful platelet depletion (**Figure 3**). In the absence of *E. coli*, NAP-2, PF4, TSP-1, TGF-β1, PDGF, and RANTES were reduced by 90% (p<0.05), 76% (p<0.001), 79% (p<0.001), 90% (p<0.001), 79% (p<0.01), and 78% (p<0.01), respectively. Addition of PCSPs significantly increased all platelet activation markers except PF4 to levels similar to, or exceeding, those observed in whole blood. The presence of *E. coli* did not substantially affect platelet activation marker levels. A general trend toward increased levels of platelet activation markers was observed across the investigated storage time points, although with different patterns.

**Figure 3:**
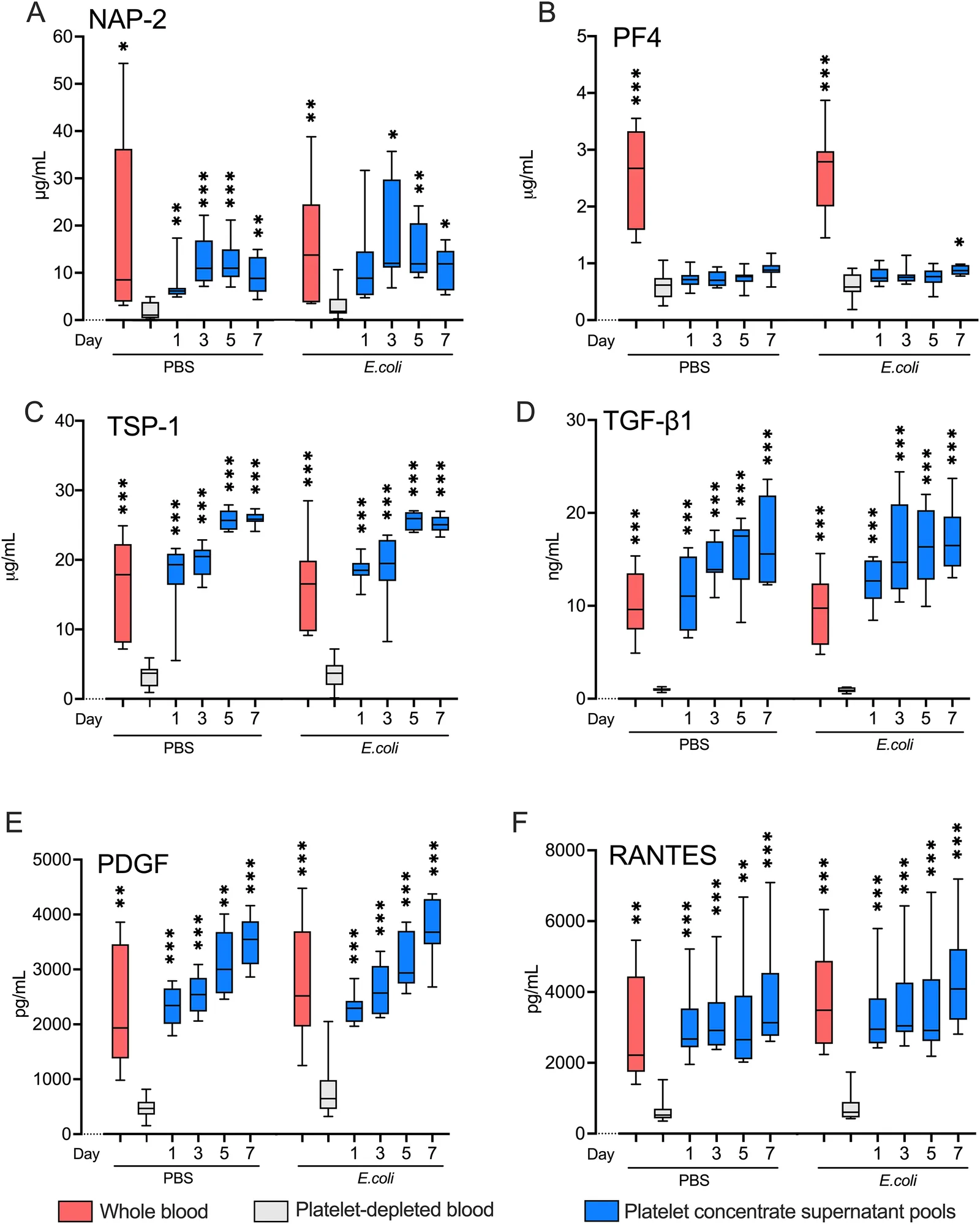
Platelet activation in platelet-depleted blood supplemented with supernatants from platelet concentrates (PCSPs). Whole blood (red), platelet-depleted blood (gray) and platelet-depleted blood supplemented with supernatants from platelet concentrates (blue) stored for 1, 3, 5 and 7 days was incubated for four hours at 37 °C with PBS or *E. coli* (10^7^/mL), as indicated. Platelet activation was analyzed as the release of NAP-2 **(A)**, PF4 **(B)**, TSP-1 **(C)**, TGF-β1 **(D)**, PDGF **(E)**, and RANTES **(F)**. Data are shown in box (25th to 75th percentiles) and whiskers (min to max) plots, (n=8). Differences between whole blood and platelet-depleted blood were analyzed with a paired Student´s *t-test*. Any effects of PCSP addition were analyzed with a repeated measures one-way ANOVA with Dunnett’s correction for multiple tests using platelet-depleted blood as the control. Statistical significance is shown as *p<0.05, **p<0.01, ***p<0.001.

### 3.3 PCSP addition to platelet-depleted blood differentially altered growth factor release

Four growth factors (FGF, G-CSF, GM-CSF, and VEGF) were quantified (**Figure 4**). Under non-*E. coli* conditions, only FGF (**Figure 4A**) and G-CSF (**Figure 4B**) increased following addition of PCSPs, with FGF increasing 2.3-3.3-fold (p<0.01-0.001) and G-CSF 1.7-3.2-fold (p<0.05). Under *E. coli* stimulation, all growth factors were significantly lower in platelet-depleted blood than in whole blood, with reductions ranging from 31-38% (p<0.01-0.001). *E. coli* induced a marked increase in growth factor release, corresponding to 6.7-15-fold increases in whole blood and 47-105-fold increases in platelet-depleted blood. Furthermore, lower G-CSF (22-23% reduction, p<0.05; **Figure 4B**) and GM-CSF (31-36% reduction, p<0.01; **Figure 4C**) responses were observed following addition of PCSPs obtained after five or seven days of storage, whereas no apparent differences were observed with PCSPs obtained after one to three days of storage.

**Figure 4:**
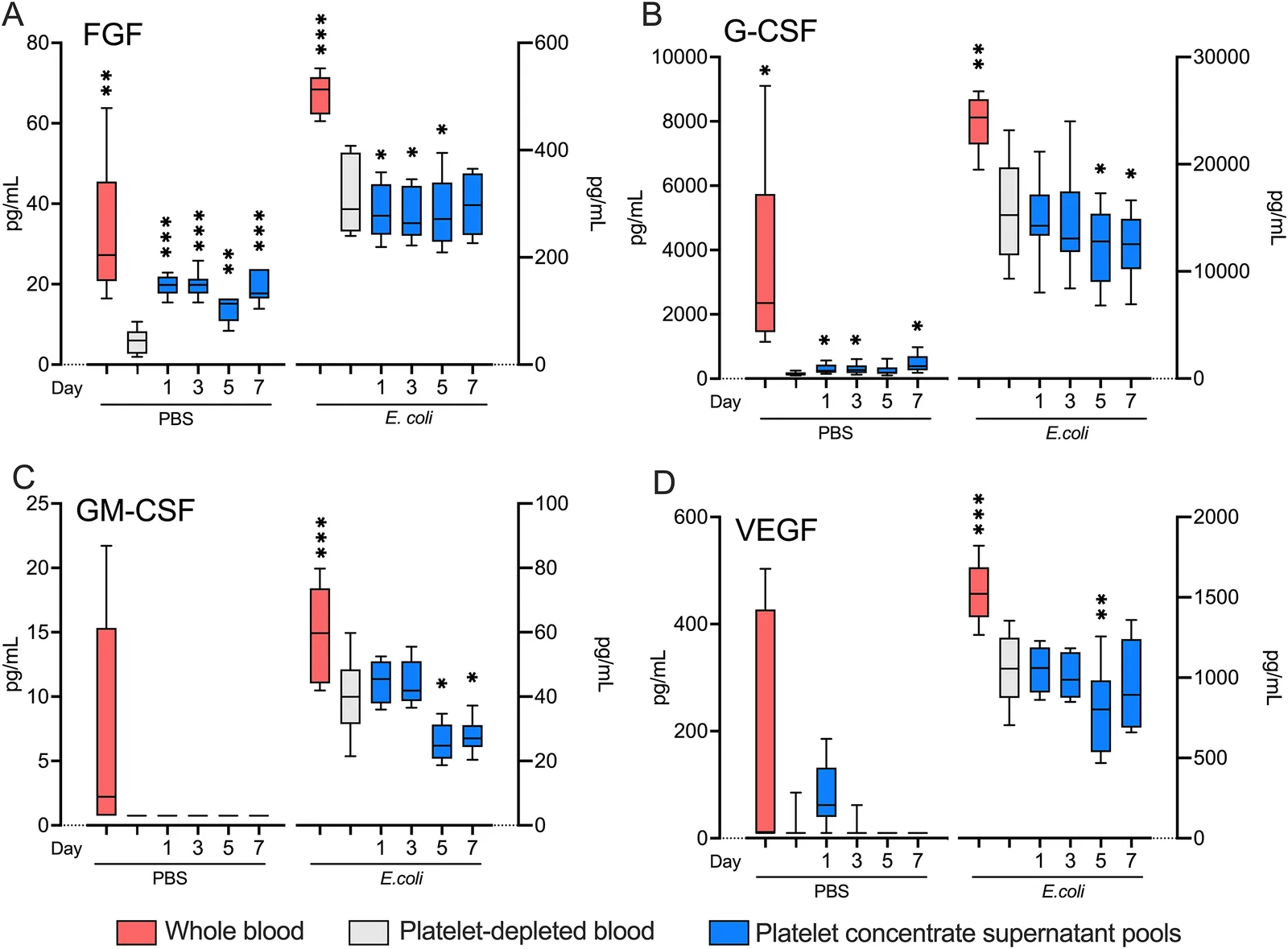
Growth factors in platelet-depleted blood supplemented with supernatants from platelet concentrates (PCSPs). Whole blood (red), platelet-depleted blood (gray) and platelet-depleted blood supplemented with supernatants from platelet concentrates (blue) stored for 1, 3, 5 and 7 days was incubated for four hours at 37 °C with PBS or *E. coli* (10^7^/mL), as indicated. FGF **(A)**, G-CSF **(B)**, GM-CSF **(C)** and VEGF **(D)** were quantified by a 27-plex using a Luminex MagPix system. Due to the large difference in response magnitude between PBS and *E. coli* conditions, separate y-axes were used. PBS data are shown on the left y-axis and *E. coli* data on the right y-axis. Data are shown in box (25th to 75th percentiles) and whiskers (min to max) plots, (n=8). Differences between whole blood and platelet-depleted blood were analyzed with a paired Student´s *t-test*. Any effects of PCSP addition were analyzed with a repeated measures one-way ANOVA with Dunnett’s correction for multiple tests using platelet-depleted blood as the control. Statistical significance is shown as *p<0.05, **p<0.01, ***p<0.001.

### 3.4 PCSP addition to platelet-depleted blood had minor effect on chemokine release

All chemokine levels were lower in platelet-depleted blood compared to whole blood and were elevated in response to *E. coli* (**Figure 5**). All chemokines except IP-10 increased significantly following addition of PCSPs (p<0.05-p<0.001). Eotaxin and IL-8 increased under both non-stimulated and *E. coli*-stimulated conditions (**Figures 5A, B**), with eotaxin increasing 1.6-1.8-fold and IL-8 increasing 4.8-5.7-fold in the absence of *E. coli*. MCP-1, MIP-1α, and MIP-1β also increased following PCSP addition under non-E. *coli*-stimulated conditions, with MCP-1 showing the largest response (2.1-2.7-fold increase) (**Figures 5D–F**). In contrast, under *E. coli*-stimulated conditions these chemokines were either unchanged or reduced following PCSP addition. Differences between storage time points were minimal, with only a slight decrease in MCP-1 observed at Day 5 (17% reduction, p<0.05) (**Figure 5D**).

**Figure 5:**
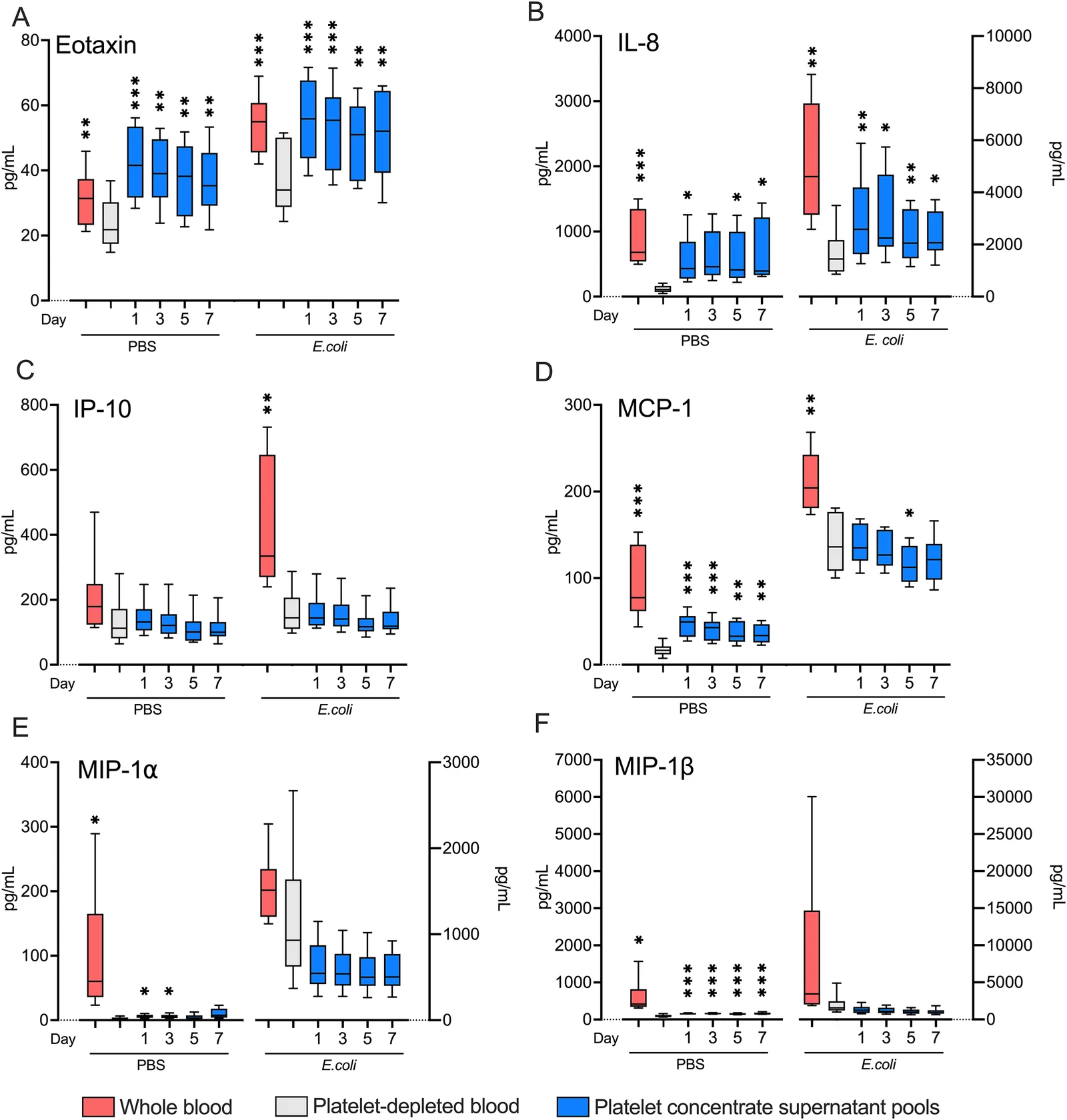
Chemokines in platelet-depleted blood supplemented with supernatants from platelet concentrates (PCSPs). Whole blood (red), platelet-depleted blood (gray) and platelet-depleted blood supplemented with supernatants from platelet concentrates (blue) stored for 1, 3, 5 and 7 days was incubated for four hours at 37 °C with PBS or *E. coli* (10^7^/mL), as indicated. Eotaxin **(A)**, IL-8 **(B),** IP-10 **(C)**, MCP-1 **(D)**, MIP-1α **(E)**, and MIP-1β **(F)** were quantified by a 27-plex using a Luminex MagPix system. Due to the large difference in response magnitude between PBS and *E. coli* conditions for IL-8, MIP-1α, MIP-1β, separate y-axes were used. PBS data are shown on the left y-axis and *E. coli* data on the right y-axis. Data are shown in box (25th to 75th percentiles) and whiskers (min to max) plots, (n=8). Differences between whole blood and platelet-depleted blood were analyzed with a paired Student´s *t-test*. Any effects of PCSP addition were analyzed with a repeated measures one-way ANOVA with Dunnett’s correction for multiple tests using platelet-depleted blood as the control. Statistical significance is shown as *p<0.05, **p<0.01, ***p<0.001.

### 3.5 PCSP addition to platelet-depleted blood differentially altered cytokine responses

Responses among the remaining biomarkers were more heterogeneous. Levels of IL-1β (**Figure 6A**), TNF (**Figure 6D**), and IL-17 (**Figure 6E**) were significantly lower in platelet-depleted blood than in whole blood under both basal and *E. coli*-stimulated conditions. In the absence of *E. coli*, IL-1β, TNF, and IL-17 were reduced by 97% (p<0.05), 63% (p<0.001), and 77% (p<0.01), respectively. Under *E. coli* stimulation, corresponding reductions were 76% (p<0.001), 70% (p<0.001), and 40% (p<0.01). In contrast, IL-6 (**Figure 6B**) and IL-12 (**Figure 6C**) were significantly reduced only under *E. coli* stimulation, by 81% (p<0.01) and 44% (p<0.001), respectively.

**Figure 6:**
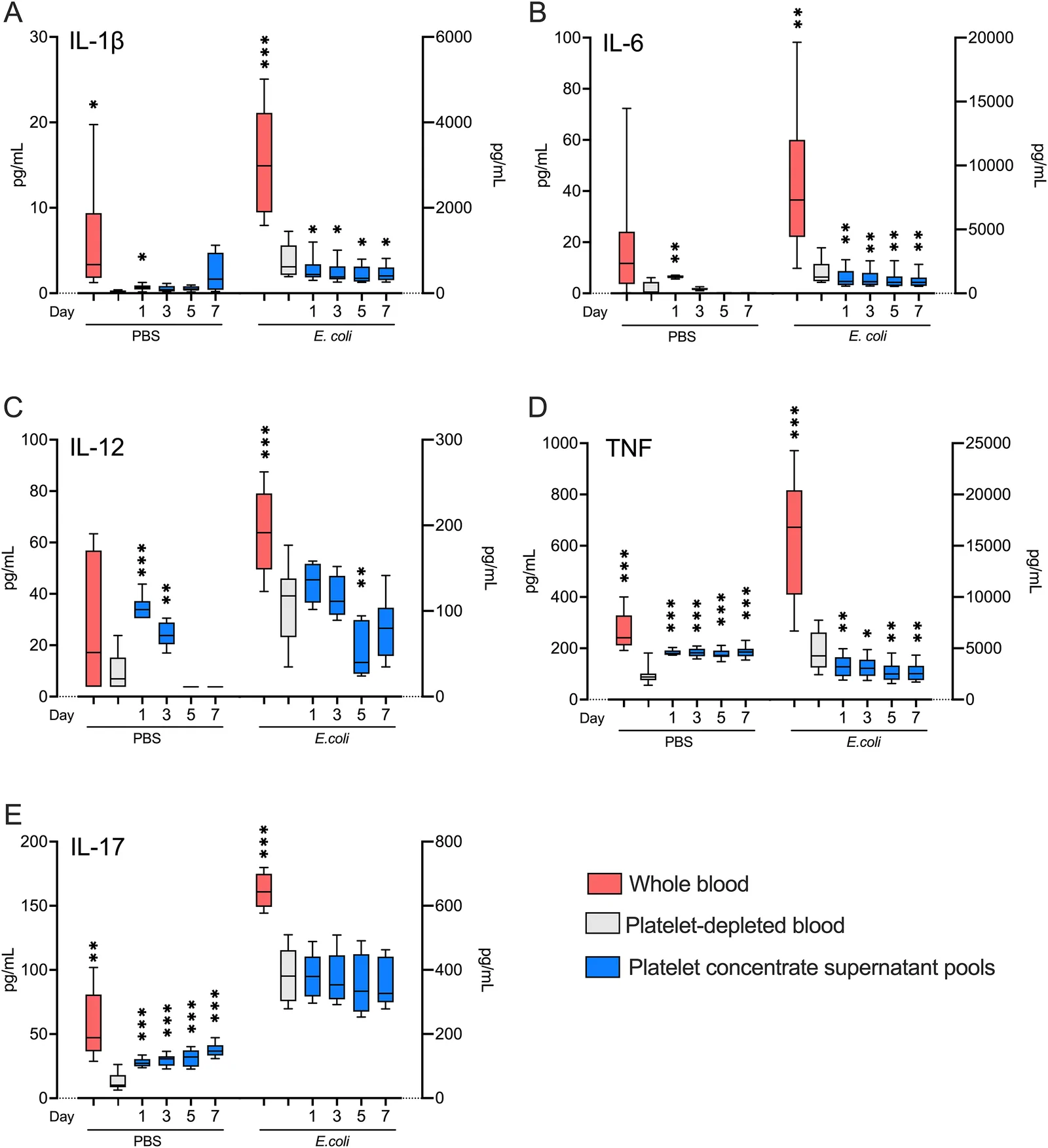
Pro-inflammatory cytokines in platelet-depleted blood supplemented with supernatants from platelet concentrates (PCSPs). Whole blood (red), platelet-depleted blood (gray) and platelet-depleted blood supplemented with supernatants from platelet concentrates (blue) stored for 1, 3, 5 and 7 days was incubated for four hours at 37 °C with PBS or *E. coli* (10^7^/mL), as indicated. IL-1β **(A)**, IL-6 **(B)**, IL-12 **(C)**, TNF **(D)** and IL-17 **(E)** were quantified by a 27-plex using a Luminex MagPix system. Due to the large difference in response magnitude between PBS and *E. coli* conditions, separate y-axes were used. PBS data are shown on the left y-axis and *E. coli* data on the right y-axis. Data are shown in box (25th to 75th percentiles) and whiskers (min to max) plots, (n=8). Differences between whole blood and platelet-depleted blood were analyzed with a paired Student´s *t-test*. Any effects of PCSP addition were analyzed with a repeated measures one-way ANOVA with Dunnett’s correction for multiple tests using platelet-depleted blood as the control. Statistical significance is shown as *p<0.05, **p<0.01, ***p<0.001.

In the absence of *E. coli*, PCSP addition significantly increased IL-1β at Day 1 (3.9-fold, p<0.05), and IL-6 at Day 1 (3.6-fold, p<0.01) (**Figures 6A, B**). A more sustained response was observed for TNF and IL-17 (**Figures 6D, E**), which increased significantly across all storage days. TNF increased 1.8-1.9-fold (p<0.001), whereas IL-17 increased 2.1-2.9-fold (p<0.001). IL-12 increased significantly following addition of Day 1 (3.7-fold, p<0.001) and Day 3 (2.5-fold, p<0.01) PCSPs, but not Day 5 or Day 7 PCSPs (**Figure 6C**). In contrast, under *E. coli*-stimulated conditions, PCSP addition significantly dampened the pro-inflammatory cytokine response (**Figures 6A, B, D**), reducing IL-1β by 25-42% (p<0.05), IL-6 by 27-39% (p<0.01), and TNF by 31-43% (p<0.05-p<0.01) across all storage days. Differences between storage time points were observed for IL-12 (**Figure 6C**), with lower responses following addition of PCSPs obtained only at Day 5 of storage (51% reduction, p<0.01).

In the presence of *E. coli*, all other cytokines (IL-1Ra, IL-2, IL-4, IL-5, IL-9, IL-10, IL-13, IL-15, and IFN-γ) were significantly lower in platelet-depleted blood than in whole blood (p<0.01-p<0.001) (**Figure 7**). In the absence of *E. coli*, IL-1Ra, IL-4, IL-9, IL-10, IL-13, and IFN-γ were also significantly lower in platelet-depleted blood (p<0.05-p<0.001). Addition of PCSPs had only minor effects on most of these cytokines. The largest effects were observed for IL-2 and IL-4 which were reduced between 16-40% (p<0.05-p<0.01) and 10-28% (p<0.01-p<0.001) respectively. Differences between storage time points were observed for IL-5 and IL-15, with lower responses following addition of PCSPs obtained after 5–7 days of storage. IL-5 was reduced by 24-29% (p<0.01), whereas IL-15 was reduced by 28-33% (p<0.05). No consistent storage-related differences were observed for the remaining cytokines.

**Figure 7:**
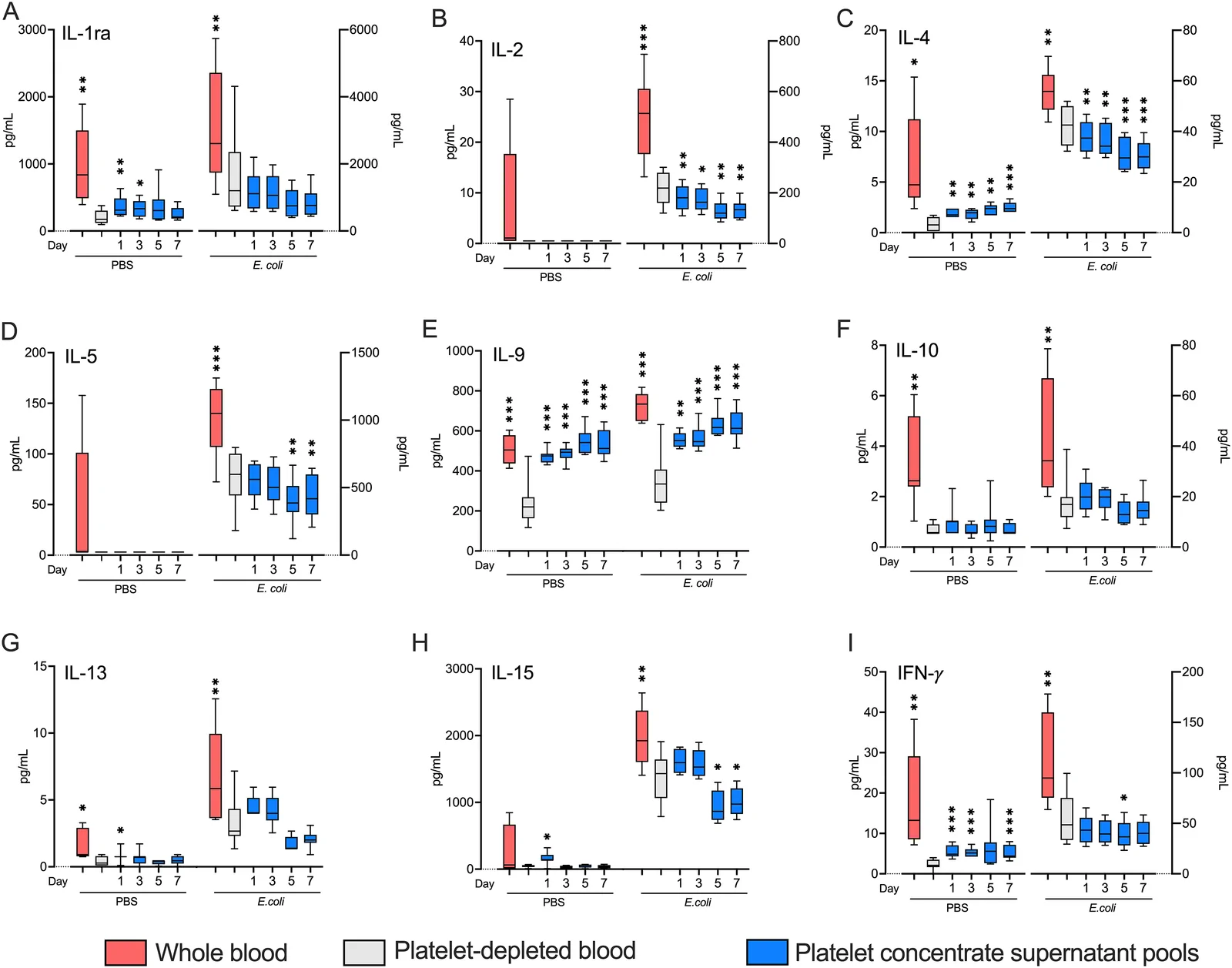
Cytokines in platelet-depleted blood supplemented with supernatants from platelet concentrates (PCSPs). Whole blood (red), platelet-depleted blood (gray) and platelet-depleted blood supplemented with supernatants from platelet concentrates (blue) stored for 1, 3, 5 and 7 days was incubated for four hours at 37 °C with PBS or *E. coli* (10^7^/mL), as indicated. IL-1ra **(A)**, IL-2 **(B)**, IL-4 **(C)**, IL-5 **(D)**, IL-9 **(E)**, IL-10 **(F)**, IL-13 **(G)**, IL-15 **(H)**, and IFN-γ **(I)** were quantified by a 27-plex using a Luminex MagPix system. Due to the large difference in response magnitude between PBS and *E. coli* conditions for IL-1ra, IL-2, IL-4, IL-5, IL-10, and IFN-γ, separate y-axes were used. PBS data are shown on the left y-axis and *E. coli* data on the right y-axis. Data are shown in box (25th to 75th percentiles) and whiskers (min to max) plots, (n=8). Differences between whole blood and platelet-depleted blood were analyzed with a paired Student´s *t-test*. Any effects of PCSP addition were analyzed with a repeated measures one-way ANOVA with Dunnett’s correction for multiple tests using platelet-depleted blood as the control. Statistical significance is shown as *p<0.05, **p<0.01, ***p<0.001.

## 4 Discussion

Platelet transfusions are frequently administered in patients with underlying inflammatory conditions, where transfused components may interact with ongoing immune activation. In this context, the present study examined how soluble factors derived from stored platelet concentrates influence inflammatory responses in a controlled ex vivo model.

We and others have previously shown that platelet concentrates accumulate increased levels of DAMPs, complement activation fragments, and platelet-derived mediators during storage (21, 22, 35). In the present study, we found that soluble factors present in PCSPs were associated with context-dependent immunomodulatory effects. In the absence of *E. coli* stimulation, the predominant effect of PCSP addition was increased production of inflammatory mediators. Excluding platelet-derived factors already present in the concentrates (22), PCSP addition induced *de novo* production of G-CSF, the chemokines IL-8 and MCP-1, as well as the immunomodulatory cytokines IL-4, IL-17, TNF, and IFN-γ. Several of these mediators are involved in leukocyte recruitment, activation, and intercellular communication, suggesting that soluble factors present in platelet concentrate supernatants trigger secondary activation of leukocytes within the recipient blood compartment.

In line with previous observations of storage lesions (21, 22, 35), we observed increased levels of complement activation products and platelet-derived factors in the PCSPs at later storage time points. At the same time, most non-platelet-derived biomarkers showed similar responses across the investigated storage time points after blood incubation, and only a limited number of cytokines, including G-CSF, GM-CSF, IL-12, and IL-15, showed lower responses at later storage time points in the presence of *E. coli*. Importantly, storage-associated changes in the molecular composition of platelet concentrates and inflammatory responses elicited by these products in recipient blood represent distinct biological readouts. Thus, the absence of a pronounced storage-duration effect on recipient cytokine responses does not exclude concomitant storage-dependent changes within the platelet concentrate itself. Furthermore, this does not imply that storage duration is biologically unimportant in general; rather, under the specific conditions examined in this exploratory study, differences between storage time points were not a dominant determinant of the observed inflammatory response.

Under *E. coli*-stimulated conditions, several key cytokines were attenuated following PCSP addition, in contrast to the non-*E. coli* condition. However, the attenuation was selective rather than global, underscoring the heterogeneous nature of the response. IL-1β, IL-2, IL-4, IL-6, and TNF showed reduced responses following PCSP addition in the presence of *E. coli*, whereas several other mediators were unchanged or increased. The potent pro-inflammatory IL-1β, IL-6, and TNF were reduced by 25-43%, while IL-2 and IL-4 were reduced by 10-40%. Although these reductions were only partial, IL-1β, IL-6, and TNF are key mediators of innate immune activation and amplification of downstream inflammatory pathways, raising the possibility that partial attenuation could influence downstream inflammatory signaling. Notably, these cytokines, together with IL-2 and IL-4, have previously been shown to be dependent on monocytes in the present whole blood model using *E. coli* as the stimulus (28), suggesting that the observed attenuation may reflect altered monocyte activation. Previous studies have demonstrated that platelet-derived mediators can both suppress and stimulate monocyte inflammatory responses depending on the signaling pathway involved. For example, platelet-derived prostaglandin E_2_, generated upon platelet activation (36), has been reported to attenuate monocyte and macrophage production of TNF and IL-6 (37, 38), whereas platelet-derived mediators, including RANTES and CD40L, together with P-selectin/PSGL-1-mediated platelet–leukocyte interactions, may promote production of inflammatory chemokines such as IL-8 and MCP-1 (30, 39–41). Excluding mediators already present in the platelet concentrate supernatants, IL-8 was the most prominently induced cytokine in recipient blood following PCSP exposure. The simultaneous presence of multiple platelet-derived mediators within the platelet concentrate supernatants may therefore contribute to selective modulation of distinct inflammatory pathways rather than a uniform anti- or pro-inflammatory effect. At the same time, the present ex vivo model was not designed to assess clinical outcomes, and the observed cytokine attenuation should therefore not be interpreted as evidence of a clinically meaningful reduction in transfusion-related inflammatory complications, and should therefore be regarded as exploratory and hypothesis-generating.

The attenuation of selected pro-inflammatory cytokines under stimulated conditions is in line with previous experimental studies showing that platelets and platelet-derived factors can dampen cytokine responses in LPS-stimulated immune cells (38, 42, 43). However, these findings should be interpreted in the context of the simultaneous pro-inflammatory effects observed under non-stimulated conditions, indicating that soluble factors present in platelet concentrates can exert both potentiating and inhibitory effects depending on the inflammatory context. This duality is consistent with the broader literature, where platelet transfusion has been associated with both protective and detrimental inflammatory outcomes, including contributions to transfusion-related complications such as TRALI (44).

Heat-inactivated *E. coli* was used as a standardized innate immune stimulus due to its dual ability to initiate toll-like receptor signaling, particularly through LPS-induced activation of TLR4, as well as to initiate complement activation pathways (29, 45). The relatively modest complement response observed suggests that the inflammatory signaling in this setup was predominantly TLR-driven. This observation aligns with the lower levels of complement-dependent cytokines, such as IL-8 and MCP-1 (46), compared to TLR4-induced cytokines, including IL-6 and TNF (47). Thus, TLR4 activation appears to play a significant role in mediating inflammatory responses in this model, but this represents a limitation, as it does not capture the full spectrum of clinically relevant inflammatory triggers, and responses to other stimuli may differ. Alternative stimuli, such as purified LPS, Gram-positive bacteria, or TLR2 agonists, engage distinct pattern-recognition pathways and may therefore produce different immunomodulatory responses. The context-dependent effects observed in the present study suggest that both the magnitude and direction of platelet supernatant-mediated modulation may depend on the nature of the inflammatory stimulus. Future studies will be required to determine whether similar effects are observed in models dominated by other innate immune pathways.

The molecular composition and immunomodulatory activity of platelet concentrates are influenced not only by storage-related changes and the accumulation of platelet-derived mediators, cytokines, and complement activation products, but also by extrinsic processing variables, including centrifugation protocols, anticoagulants, storage conditions, and collection and manufacturing procedures (48–50). Furthermore, intrinsic donor-related factors, including age, sex, baseline platelet characteristics, health status, and other biological variables, may contribute to product heterogeneity (48, 49, 51). Together, these observations highlight the importance of considering both intrinsic and extrinsic sources of variability when evaluating the immunomodulatory properties of platelet-derived products.

Previous studies have reported storage-associated accumulation of IL-6 and IL-8 in platelet concentrates, particularly in products with higher residual leukocyte content, whereas pre-storage leukoreduction markedly reduces this accumulation (52–55). Differences in product composition and processing may therefore contribute to differences in the storage-associated cytokine profile between historical platelet products and contemporary leuko-reduced platelet concentrates used in the present study.

Several limitations should be acknowledged. First, the study included six platelet concentrates that, for practical and methodological reasons, were pooled according to storage day before use in the incubation experiments. While this approach enabled assessment of the biological effects of platelet concentrate supernatants across multiple recipient blood donors, it precluded assessment of variability between individual platelet concentrate units. Consequently, storage-time related findings should be interpreted with caution and regarded as exploratory and hypothesis-generating rather than definitive evidence of storage-dependent biological effects. Several of the biomarkers investigated in the present study have previously been characterized in individual platelet concentrates of the same production type and showed relatively limited inter-unit variability (22). Future studies including larger numbers of individual platelet concentrates will be needed to better define donor-to-donor variability and strengthen the generalizability of storage-related findings. Second, a single PCSP concentration (25% v/v) was used throughout the study. This concentration was selected to approximate the platelet-derived mediator exposure corresponding to restoration of the platelet concentration toward the physiological range, while ensuring sufficient biological signal detection in the ex vivo model. However, the exposure achieved in the present setup is higher than that obtained following a single platelet transfusion. Consequently, the magnitude of the observed responses should not be interpreted as a direct estimate of clinical effect size.

Third, while necessary for isolating platelet-derived effects, the use of platelet-depleted blood resulted in partial loss of leukocytes, complicating direct comparisons with whole blood responses. This protocol was selected because it achieves extensive platelet depletion while preserving leukocyte responsiveness and minimizing artefactual cellular activation (30). Consequently, some of the differences observed between whole blood and platelet-depleted blood may reflect both platelet depletion and reduced numbers of responding leukocytes. However, the primary analyses of platelet concentrate supernatant effects were performed within the platelet-depleted blood system, where leukocyte numbers were comparable between experimental conditions and therefore less likely to account for the observed effects of PCSP supplementation. Fourth, the study focuses exclusively on soluble mediators present in platelet concentrate supernatants and therefore does not capture direct interactions between intact platelets and leukocytes. Such interactions include CD40L-CD40 signaling, P-selectin/PSGL-1-mediated cell-cell communication, platelet-leukocyte aggregate formation, and other receptor-dependent pathways known to modulate inflammatory responses (56). Consequently, the observed effects should be interpreted as reflecting the biological activity of soluble storage-derived factors rather than the full immunomodulatory potential of transfused platelet concentrates.

## 5 Conclusions

Taken together, these results suggest that soluble factors released from stored platelets can modulate inflammatory responses in an ex vivo setting, but that the direction and magnitude of the effect depend strongly on the inflammatory context. Under basal conditions the effect was predominantly pro-inflammatory, whereas under *E. coli* stimulation the response was more mixed and included attenuation of selected cytokines. The effects of platelet concentrates on inflammatory response were therefore not uniformly pro- or anti-inflammatory, but context dependent and showed generally similar recipient-cell responses across the investigated storage-day pools.

## Data Availability

The raw data supporting the conclusions of this article will be made available by the authors, without undue reservation
