# Supplementary material for: Supernatants from stored platelet concentrates modulate the inflammatory response in platelet-depleted blood ex vivo
Source: Front Immunol. 2026 Oct 5;17:1928875. doi: 10.3389/fimmu.2026.1928875 (PMC13677499; doi:10.3389/fimmu.2026.1928875)
Supplement: Supplementary file 1 [file Table1.docx]

**Supplementary Table S1.** Assay performance characteristics for the respective biomarkers analyzed.

| **Analyte** | **Platform** | **LLOQ^1,2^** | **ULOQ^1,3^** | **LOD^4^** | **Intra-assay**  **CV (%)^1^** | **Inter-assay**  **CV (%)^1^** | **Dilution factor** |
| --- | --- | --- | --- | --- | --- | --- | --- |
| C3bc | ELISA | 0.02 (CAU/mL) | 20 (CAU/mL) | 0.02 (CAU/mL) | 7 | 8 | 150 |
| TCC | ELISA | 0.06 (CAU/mL) | 20 (CAU/mL) | 0.06 (CAU/mL) | 5 | 5 | 5 |
| NAP-2 | ELISA | 15.6 (pg/mL) | 1000 (pg/mL) | 15.6 (pg/mL) | N.D.^5^ | N.D. | 10000-100000 |
| PF4 | ELISA | 15.6 (pg/mL) | 1000 (pg/mL) | 15.6 (pg/mL) | N.D. | N.D. | 10000 |
| TSP-1 | ELISA | 1560 (pg/mL) | 100000 (pg/mL) | 1560 (pg/mL) | N.D. | N.D. | 1000 |
| TGF-β1 | ELISA | 31.2 (pg/mL) | 2000 (pg/mL) | 31.2 (pg/mL) | N.D. | N.D. | 10 |
| IL-1β | Luminex | 3.2 (pg/mL) | 3261 (pg/mL) | 0.3 (pg/mL) | 6 | 8 | 4 |
| IL-1Ra | Luminex | 81.1 (pg/mL) | 70487 (pg/mL) | 36.8 (pg/mL) | 9 | 8 | 4 |
| IL-2 | Luminex | 2.1 (pg/mL) | 17772 (pg/mL) | 1.0 (pg/mL) | 7 | 9 | 4 |
| IL-4 | Luminex | 2.2 (pg/mL) | 3467 (pg/mL) | 0.3 (pg/mL) | 9 | 8 | 4 |
| IL-5 | Luminex | 3.1 (pg/mL) | 7380 (pg/mL) | 6.2 (pg/mL) | 8 | 10 | 4 |
| IL-6 | Luminex | 2.3 (pg/mL) | 18880 (pg/mL) | 0.4 (pg/mL) | 7 | 11 | 4 |
| IL-8 | Luminex | 1.9 (pg/mL) | 26403 (pg/mL) | 0.9 (pg/mL) | 9 | 4 | 4 |
| IL-9 | Luminex | 2.1 (pg/mL) | 7989 (pg/mL) | 1.7 (pg/mL) | 8 | 9 | 4 |
| IL-10 | Luminex | 2.2 (pg/mL) | 8840 (pg/mL) | 1.1 (pg/mL) | 5 | 6 | 4 |
| IL-12 | Luminex | 3.3 (pg/mL) | 13099 (pg/mL) | 7.6 (pg/mL) | 6 | 6 | 4 |
| IL-13 | Luminex | 3.7 (pg/mL) | 3137 (pg/mL) | 0.2 (pg/mL) | 8 | 7 | 4 |
| IL-15 | Luminex | 2.1 (pg/mL) | 2799 (pg/mL) | 19.5 (pg/mL) | 5 | 6 | 4 |
| IL-17 | Luminex | 4.9 (pg/mL) | 12235 (pg/mL) | 2.7 (pg/mL) | 8 | 6 | 4 |
| Eotaxin | Luminex | 40.9 (pg/mL) | 5824 (pg/mL) | 0.1 (pg/mL) | 8 | 11 | 4 |
| FGF | Luminex | 27.2 (pg/mL) | 7581 (pg/mL) | 4.3 (pg/mL) | 8 | 8 | 4 |
| G-CSF | Luminex | 2.4 (pg/mL) | 11565 (pg/mL) | 7.5 (pg/mL) | 10 | 5 | 4 |
| GM-CSF | Luminex | 63.3 (pg/mL) | 6039 (pg/mL) | 1.5 (pg/mL) | 12 | 6 | 4 |
| IFN-γ | Luminex | 92.6 (pg/mL) | 52719 (pg/mL) | 1.1 (pg/mL) | 15 | 9 | 4 |
| IP-10 | Luminex | 18.8 (pg/mL) | 26867 (pg/mL) | 1.7 (pg/mL) | 11 | 9 | 4 |
| MCP-1 | Luminex | 2.1 (pg/mL) | 1820 (pg/mL) | 2.0 (pg/mL) | 9 | 7 | 4 |
| MIP-1α | Luminex | 1.4 (pg/mL) | 836 (pg/mL) | 0.2 (pg/mL) | 7 | 8 | 4 |
| MIP-1β | Luminex | 2.0 (pg/mL) | 1726 (pg/mL) | 1.9 (pg/mL) | 8 | 8 | 4 |
| PDGF | Luminex | 7.0 (pg/mL) | 51933 (pg/mL) | 3.7 (pg/mL) | 9 | 8 | 4 |
| RANTES | Luminex | 2.2 (pg/mL) | 8617 (pg/mL) | 1.1 (pg/mL) | 9 | 6 | 4 |
| TNF | Luminex | 5.8 (pg/mL) | 95484 (pg/mL) | 3.1 (pg/mL) | 8 | 6 | 4 |
| VEGF | Luminex | 5.5 (pg/mL) | 56237 (pg/mL) | 20.0 (pg/mL) | 9 | 7 | 4 |

^1^ Data for C3bc and TCC retrieved from Bergseth et al. 2013(1); data for NAP-2, PF4, TSP-1, TGF-β1 was retrieved from respective assays protocol from the manufacturer (R&D Systems, Minneapolis, MN); data for all other markers retrieved from assays protocol from the manufacturer (Bio-Rad, Hercules, CA)

² Lower Limit of Quantification

^3^ Upper Limit of Quantification

^4^ Operational limit of detection (LOD) used in the present study.

^5^ N.D., not determined

1. Bergseth G, Ludviksen JK, Kirschfink M, Giclas PC, Nilsson B, Mollnes TE. An International Serum Standard for Application in Assays to Detect Human Complement Activation Products. *Mol Immunol* (2013) 56(3):232-9. Epub 20130617. doi: 10.1016/j.molimm.2013.05.221.
